# Head removal enhances planarian electrotaxis

**DOI:** 10.1242/jeb.243972

**Published:** 2022-09-07

**Authors:** Ziad Sabry, Rui Wang, Aryo Jahromi, Christina Rabeler, William B. Kristan, Eva-Maria S. Collins

**Affiliations:** 1 Department of Biology, Swarthmore College, Swarthmore, PA 19081, USA; 2 Department of Bioengineering, University of California San Diego, La Jolla, CA 92037, USA; 3 Department of Mechanical Engineering, University of California San Diego, La Jolla, CA 92037, USA; 4 Department of Biological Sciences, California State University San Marcos, San Marcos, CA 92096, USA; 5 Department of Physics and Astronomy, Swarthmore College, Swarthmore, PA 19081, USA; 6 Department of Physics, University of California San Diego, La Jolla, CA 92037, USA

**Keywords:** *Dugesia japonica*, Behavior, Galvanotaxis, Locomotion, Decapitation

## Abstract

Certain animal species utilize electric fields for communication, hunting and spatial orientation. Freshwater planarians move toward the cathode in a static electric field (cathodic electrotaxis). This planarian behavior was first described by Raymond Pearl more than a century ago. However, planarian electrotaxis has received little attention since, and the underlying mechanisms and evolutionary significance remain unknown. To close this knowledge gap, we developed an apparatus and scoring metrics for automated quantitative and mechanistic studies of planarian behavior upon exposure to a static electric field. Using this automated setup, we characterized electrotaxis in the planarian *Dugesia japonica* and found that this species responds to voltage instead of current, in contrast to results from previous studies using other planarian species. Surprisingly, we found differences in electrotaxis ability between small (shorter) and large (longer) planarians. To determine the cause of these differences, we took advantage of the regenerative abilities of planarians and compared electrotaxis in head, tail and trunk fragments of various lengths. We found that tail and trunk fragments electrotaxed, whereas head fragments did not, regardless of size. Based on these data, we hypothesized that signals from the head may interfere with electrotaxis when the head area/body area reached a critical threshold. In support of this hypothesis, we found that (1) smaller intact planarians that cannot electrotax have a relatively larger head-to-body-ratio than large planarians that can electrotax, and (2) the electrotaxis behavior of cut head fragments was negatively correlated with the head-to-body ratio of the fragments. Moreover, we could restore cathodic electrotaxis in head fragments via decapitation, directly demonstrating inhibition of electrotaxis by the head.

## Abstract (precis)

**Summary:** A new method for quantitative studies of planarian electrotaxis shows that *Dugesia japonica* move toward the cathode. This behavior is enhanced by removal of the head.

## INTRODUCTION

Freshwater planarians are soft-bodied flatworms famous for their regenerative abilities (Rink, 2018). Planarians have a large repertoire of behaviors that can be used as readouts of brain function (Inoue et al., 2015; Zhang et al., 2019). Over a century ago, Raymond Pearl (1903) was the first to write a comprehensive description of planarian behaviors, including ciliary-driven gliding and musculature-driven locomotion (peristalsis and scrunching), phototaxis, chemotaxis and thermotaxis. Recently, planarians have experienced a resurgence as a neurobiology system because modern molecular biology techniques paired with computer vision now allow for mechanistic and quantitative studies of their behavior. For example, it was shown that ciliary gliding depends on serotonergic signaling (Currie and Pearson, 2013), that peristalsis and scrunching are distinct gaits (Cochet-Escartin et al., 2015), with peristalsis resulting from non-functional cilia (Rompolas et al., 2010) and scrunching being a cilia-independent escape gait (Cochet-Escartin et al., 2015). Thermotaxis, phototaxis and chemotaxis have been found to require the presence of a brain to sense their respective stimuli (Inoue et al., 2015), whereas fission (Malinowski et al., 2017; Goel et al., 2021), scrunching (Cochet-Escartin et al., 2015) and avoidance of local near-ultraviolet light stimulation (Paskin et al., 2014; Shettigar et al., 2017; Le et al., 2021; Shettigar et al., 2021) can occur without a brain.

Here, we characterize electrotaxis, another planarian behavior which was first described by Pearl over a century ago (Pearl, 1903) but, to the best of our knowledge, has not been rigorously revisited. Pearl (1903) showed that members of various planarian species (*Planaria maculata*, *Planaria dorotocephala* and *Planaria gonocephala*; Table S1) turn toward the negatively charged electrode (cathode) when an electrical field is applied. He observed that the end of the planarian closest to the positively charged electrode (anode) contracted, comparable to the response observed by mechanical stimulation. Pearl interpreted this as evidence of the current acting directly on the muscles rather than interacting with sensory organs or cilia. Furthermore, he reported that planarians became ‘wholly or partially paralyzed in a very short time after the current begins to act, and as a consequence the reactions become feeble and indistinct’ (Pearl, 1903). Unfortunately, no information on the duration of these experiments was provided, but this description of planarian behavior suggests the use of strong electric fields. Finally, Pearl found that head pieces from transversely cut planarians behaved identically to intact worms, whereas tail pieces displayed contraction on the anode-facing end but did not reorient or move toward the cathode.

Subsequent studies in the first half of the 20th century by a handful of researchers on various planarian species (Table S1) confirmed that intact planarians either oriented and moved toward the cathode (Hyman and Bellamy, 1922; Robertson, 1927; Hyman, 1932) or assumed a U or W shape, which allowed them to bring their head and, for certain species, future heads at fission locations closest to the cathode, depending on the strength of the electric field (Hyman and Bellamy, 1922; Hyman, 1932). For *Dugesia tigrina*, Pearl's observation that the end of the planarian nearest to the anode appeared to contract was confirmed (Hyman and Bellamy, 1922; Robertson, 1927; Hyman, 1932). However, in contrast to Pearl's findings, all planarian fragments (heads, trunks and tails) were reported to exhibit cathodic electrotaxis (Robertson, 1927; Fries, 1928; Marsh and Beams, 1952; Viaud, 1952a).

Different mechanisms have been proposed to explain planarian electrotaxis: (1) direct action of electrical current on nerve or muscle cells (Pearl, 1903; Fries, 1928); (2) an intrinsic bioelectric gradient in the body of the animal, with a positively charged head and a negatively charged tail (Hyman and Bellamy, 1922; Robertson, 1927; Hyman, 1932); (3) a bioelectric gradient with a negatively charged head and a positively charged tail that causes electrophoresis of a negatively charged diffusible head inhibitor molecule with the source at the head (Lange and Steele, 1978); or (4) directional differences in conductance (with less resistance in the head) and excitation along the anterior–posterior axis (Viaud and Medioni, 1951; Viaud, 1952a,b). The existing experimental data, however, are insufficient to distinguish among these theories. Furthermore, because different researchers used different planarian species, varying experimental conditions and manual scoring metrics, which were rarely described in detail and may have suffered from experimenter bias (reviewed by Jenkins, 1967 and summarized in Table S1), it is difficult to compile and interpret these previous findings. Therefore, we decided to revisit planarian electrotaxis with modern experimental tools and a quantitative and automated approach using *Dugesia japonica*, a popular species for planarian behavioral studies (Shomrat and Levin, 2013; Inoue et al., 2014, 2015; Sabry et al., 2019; Zhang et al., 2019; Ireland et al., 2020; Le et al., 2021). Experiments were conducted to test how various anatomical structures affect electrotaxis, to begin to differentiate between the proposed mechanisms.

## MATERIALS AND METHODS

### Animal care

Asexual *Dugesia japonica* Ichikawa & Kawakatsu 1964 planarians were used for electrotaxis experiments. Planarians were kept in plastic containers filled with 0.5 g l^−1^ Instant Ocean (IO) water (Spectrum Brands, Blacksburg, VA, USA) and stored at 18–20°C in temperature-controlled incubators (MIR-554, Panasonic, Kadoma, Osaka, Japan) in the dark when not used for experiments. Planarians were maintained following standard protocols (Dunkel et al., 2011), fed organic beef liver once a week, cleaned twice a week and starved for at least 1 week before use in experiments.

### Electrotaxis arena setup

**Fig. 1.**
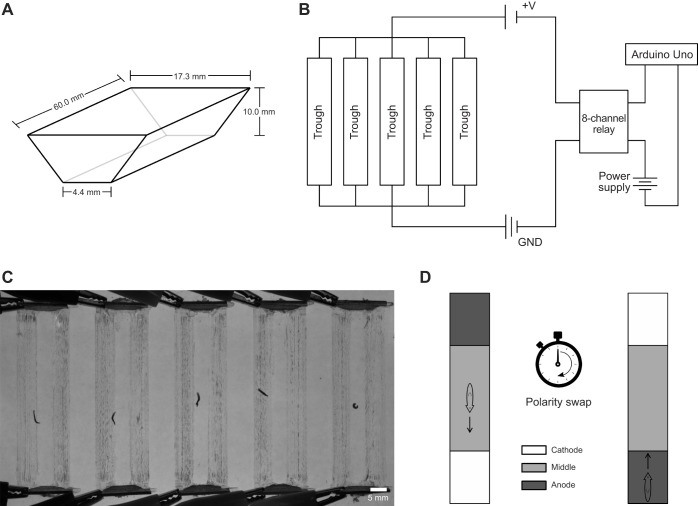
**Schematics of planarian electrotaxis setup.** (A) Schematic of one trough (arena) with an isosceles trapezoidal cross-section. (B) Circuit diagram of the electrotaxis setup. GND, ground. (C) Representative image of planarians in the arenas backlit with a red electroluminescent panel. (D) Schematic showing a planarian in an arena. Planarians were dropped in the middle of each trough at the start of the experiment. The electrical polarity was reversed after half the experiment time had elapsed. White, gray and dark gray regions denote the cathode quadrant, middle two quadrants and anode quadrant, respectively.

We developed an arena in which five planarians could be simultaneously imaged during exposure to a static electric field, with computer-controlled voltage strength and field direction. We designed a 60.0-mm-long trough arena with an isosceles trapezoidal cross-section shape. A trapezoidal shape was chosen to ensure that planarians could be observed even when moving along the container boundaries, for which they have a preference (Akiyama et al., 2015). The trough was 17.3 mm wide at the top, 4.4 mm wide at the bottom and 10.0 mm in height (Fig. 1A). Five troughs (arenas) were milled into a transparent acrylic sheet, allowing up to five independent experiments to be run simultaneously. Electrodes that took up the cross-section of the arena were cut out of a 3 mm-thick aluminum sheet and adhered with cyanoacrylate glue to either end of each arena.

The five sets of electrodes were arranged in a parallel circuit configuration. An external 18 Volt DC 2.0 Linear Bench Power Supply (Circuit Specialists, Tempe, AZ, USA) provided a voltage to the circuit. A voltage was supplied to each arena through an 8-Channel 5 V Relay Shield Module Board Optocoupler Module Arduino ARM PIC AVR [Jekewin (Amazon), Seattle, WA, USA] which was controlled by an Arduino Uno (Fig. 1B). The 8-channel relay was connected to the aluminum electrodes and to the power supply by wires with alligator clips. All other connections were made using wires on a half-size breadboard (Adafruit Industries, New York, NY, USA).

To record experiments, a Basler Ace acA640 camera (Basler AG, Ahrensburg, Germany) was mounted on a ring stand above the arenas. Images were recorded at a rate of 8 frames s^−1^ as JPEG image stacks. Circuit control via the Arduino and recording via the Basler camera were controlled through MATLAB (version R2019b, MathWorks, Natick, MA, USA). The arenas were backlit with a 20×15 cm red electroluminescent (EL) panel (Adafruit Industries) to provide contrast between the planarian and the background (Fig. 1C). The output of the EL panel was measured with a Fieldmaster power meter (Coherent, Santa Clara, CA, USA), with an average power reading of 424 nW. Using Roscolux filters (#382;89;41;32; Rosco, NY, USA), we estimated that the emission peak of the EL panel was between 600 and 700 nm, a wavelength range to which planarians are insensitive (Paskin et al., 2014; Shettigar et al., 2017) and in which they robustly respond to weaker stimuli, such as thermotaxis (Ireland et al., 2020). All experiments were conducted with the room lights turned off. When filled with IO water with an applied voltage of 2 V, the arena would experience a voltage differential of 0.33 V cm^−1^ and an approximate current density of 0.077 mA mm^−2^. IO water was measured with a conductivity meter (Traceable CC4360, VWR) to have a conductivity of ∼780 µS cm^−1^ or a resistivity of 12.8 Ωm.

### Experimental conditions

Each arena was evenly filled with 4 ml of IO water. After filling the arenas with water, a background image of the entire setup was taken to be used for later data processing. A single planarian was carefully dropped into the middle of each of the five troughs using a Samco 691 transfer pipette (Thermo Fisher Scientific, Waltham, MA, USA). Once all planarians were approximately centered in their arenas, planarians were exposed to the electric field and recorded. After half the predetermined experimental time elapsed, the electrical polarity was swapped (Fig. 1D). Recording was terminated and the voltage was brought to 0 V at the conclusion of each experiment. Planarians were subsequently removed from the arenas and placed in a recovery container. Prior to the beginning of another experiment, IO water was drained from each arena and the arenas were wiped down with a paper towel to remove any mucus trails. All experiments were conducted at room temperature. For the voltage sweep, planarians were released approximately in the middle of their troughs at experiment onset (Fig. 1D). The experiment was 120 s in duration, with a polarity swap at 60 s (three technical replicates with five planarians each in individual troughs for each voltage). To determine whether planarians responded to electrical current or to voltage, troughs were filled with 4 ml of either IO or ultrapure (Milli-Q; MQ) water. Experiments were run at 4 V for 90 s without a polarity swap. The higher voltage of 4 V was chosen to achieve either a high voltage, high current condition (using IO water) or a high voltage, low current condition (using ultrapure water).

### Temperature, convective currents and pH tests

Temperature and pH differentials were measured when a 2 V electric field was applied to the arena, filled with 4 ml of IO water, for 360 s, with a polarity swap at 180 s (Fig. S1). To measure temperature, an image of the arenas was taken before and after the electric field was applied using a FLIR infrared camera (FLIR Systems, Wilsonville, OR, USA). We measured a negligible temperature gradient of ∼0.5°C in our apparatus (which is within the 5% accuracy range of the instrument). For reference, a gradient of ∼8°C was required to induce thermotaxis in *D. japonica* (Inoue et al., 2014). The pH was measured using pH test strips (Whatman, Maidstone, UK), and was found to be approximately 6.5 both before and after the electric field was applied. To test for convection, a drop of food coloring dye (Gel Spice Company, Bayonne, NJ, USA) was placed at the initial anode before a 2 V electric field was applied to an arena for 180 s, with a polarity swap at 90 s. For comparison, a drop of food coloring dye was placed in the same region of a different arena with a 0 V electric field. An image of the arenas was taken before and after the electric field was applied to visualize the convective currents through the dye movement.

### Amputation experiments

For all experiments involving amputations, transverse amputations were used. To generate head and tail pieces, planarians were amputated either just above (pre-pharyngeal) or just below (post-pharyngeal) the pharynx using a sterile razor blade. For experiments involving trunk pieces, planarians were amputated both pre-pharyngeally and just below the auricles. For successive amputations, cuts were administered transversally to the head–tail axis in series. After each amputation, planarians were given at least 1 day to heal prior to conducting electrotaxis experiments. Because small tail pieces are less mobile than intact worms (Inoue et al., 2014, 2015), we increased the duration of the experiment when assessing post-pharyngeally cut tails. Head pieces and pre-pharyngeal tails were exposed to the electric field for 240 s, with an electrical polarity swap at 120 s; post-pharyngeal tails were exposed for 360 s with a polarity swap at 180 s.

### Raw image data processing

Raw image data was imported into Fiji (Schindelin et al., 2012) for background subtraction. The five arenas were separated into five image stacks using the rectangle tool to draw equal-sized rectangles around each arena and duplicating into individual image stacks. The arena background was subtracted from each of the five image stacks using the background image taken at the start of each experiment. The five stacks were then saved separately.

### Data analysis and statistics

Processed frames were imported into MATLAB and binarized by manually setting a threshold that encompassed only the planarian. The center of mass of each planarian was then tracked in each frame using custom MATLAB code as previously described (Talbot and Schötz, 2011). The time spent in the arena quadrants and the fraction of time spent moving toward the cathodes for each planarian were outputted and compiled into a spreadsheet. To quantify the response of the planarian to the electrical field, we calculated two parameters before (‘1’) and after (‘2’) the polarity swap. First, we calculated the fraction of time spent in the quadrant containing the cathode during the first or second half of the experiment (*f*_cat-1,2_, time spent in cathode quadrant divided by total time with cathode at that location) and the fraction of time spent moving toward the cathode (*f*_mov-1,2_). To determine the movement relative to the cathode, we only used the *y*-coordinate (one-dimensional motion). For each frame *j* in which the current center of mass (COM) coordinate *y*(*j*) is closer to the cathode position (set to *y*=0) than in the previous frame *y*(*j*–1), the planarian was scored as moving toward the cathode. The number of frames for which the planarian was scored as moving toward the cathode was then divided by the total number of frames for which the planarian was visible, yielding *f*_mov-1,2_*.* When the planarian reached the cathode, its COM was not recorded as the planarian was not visible to the program. Therefore, *f*_mov-1,2_ complemented *f*_cat-1,2_, which measures the time spent at the cathode. If a planarian moved randomly, it was expected that on average it would spend equal amounts of time moving toward and away from the cathode. We did not set any thresholds, require persistence in motion or determine the velocity of motion, to avoid introducing additional parameters in the analysis. For trunk and tail pieces, which are not as mobile and thus are less likely to reach the cathode, *f*_mov-1,2_ is the most important parameter. The fractions of experimental time spent in the middle two quadrants and in the anode quadrant (before and after the electrical polarity swap) were also recorded.

For length and area ratio measurements, the threshold method described above was used and the area and length of the body calculated using the built-in Analyze Particles function in Fiji. Head area and head length were manually measured by a researcher who was not involved in this project and thus naïve to the hypotheses, to prevent possible bias in the analysis. Ratios were calculated in MATLAB for visualization and in R (version 4.1.2, R Core Team) for statistical analysis. High magnification imaging of small and large planarians of the same size range as used for electrotaxis experiments was conducted to ensure that the analysis of the lower magnification images from the electrotaxis setup did not introduce any artifacts.

Responses to the electrical field were tested using ANOVA models. Response variables were proportions, either of trial time spent in the cathode zone or of trial time spent moving toward the cathode, before or after polarity swaps. Differences in these values between controls measured without a voltage applied and the treatment-group worms with a voltage applied constituted electrotaxis. For the initial test of electrotaxis at varying voltage levels, a one-way ANOVA was used with voltage as a predictor variable. Significant effects of voltage were followed up with Dunnett's *post hoc* tests of 0 V controls against the non-zero voltages. Experiments with additional treatments were analyzed with factorial ANOVA. As the difference between 0 and 2 V was the indication of electrotaxis, the effect of other predictors (such as worm size) on electrotaxis was indicated by a significant interaction between voltage and the other predictors (that is, the amount of electrotaxis depends on worm size if the voltage×size interaction is significant). When significant interactions were detected, the difference between the 0 V and 2 V groups at the levels of the other predictor were used as *post hoc* procedures, using Tukey's method to account for multiple comparisons. *Post hoc* comparisons were only conducted following a significant ANOVA, so *P*-values for *post hoc* comparisons of group means are reported with the *post hoc* procedure identified.

The successive cuts experiment used the same planarians with treatments that changed each day. These repeated-measures data were analyzed using the successive treatments as a within-subjects factor. The within-subjects treatments applied were different between two groups of worms, so the group was used as a between-subjects factor. The interaction of the within- and between-subjects factors indicated that the two groups differed in their responses to the two different treatment sequences. The same worms were tested at 0 and 2 V to test for electrotaxis on each day, so *post hoc* comparisons between the voltage levels were done with two-tailed paired *t*-tests, and significance was assessed with a Bonferroni-adjusted α level (six comparisons were made, so *P*-values needed to be less than 0.05/6=0.008 to be considered statistically significant for the *post hoc* two-tailed paired *t*-tests).

Proportions often violate assumptions of normality and homoscedasticity, so these assumptions were tested prior to each analysis. When one or more assumptions were violated, we used non-parametric randomization tests to confirm that the statistical significance of model terms was not affected. If significance was unchanged by using randomization tests, then *post hoc* procedures were conducted as usual, using either Tukey or Dunnett tests. All analysis was done with the R statistical computing language (version 4.1.2, https://www.r-project.org/) and extension libraries. *Post hoc* procedures were done with the library emmeans (version 1.7.0). Randomization tests were performed using the library lmPerm (2.1.0). Homoscedasticity was tested with a Breusch–Pagan test from the lmtest library (0.9-38). Repeated measures analysis was done with the car library (3.0-11). The data, analysis in R and respective figures can be downloaded from the Collinslab GitHub repository (https://github.com/Collinslab-swat/Planarian-electrotaxis).

## RESULTS

### *Dugesia japonica* exhibits cathodic electrotaxis at 2 V without overt adverse effects

To determine what field strength was necessary to induce electrotaxis, we conducted a voltage sweep (Table 1; Fig. S2). At 0 V, planarians moved randomly and one would have expected them to spend 25% of the experimental time in each quadrant. We found that they spent approximately one-fourth to one-third of the experimental time in the quadrants containing the cathode (Table 1; Fig. S2). The increased time spent in the quadrants containing the electrodes compared with the two middle quadrants likely resulted from planarians exhibiting wall preference (Akiyama et al., 2015), as the electrode-containing quadrants have more walls than the middle two quadrants (Fig. 1C,D). Planarians did not exhibit a preference for movement toward either electrode at 0 V; they moved toward and away from the cathode before and after polarity swap at equal rates (Table 1; Fig. S2, Movie 1). When an electric field of 1 V was applied, planarians spent more time moving toward and staying in the cathode-containing quadrants, but the increase was not significant compared with what was seen at 0 V (Dunnett's test, *P*=0.587). At 1.5–2 V, planarians reoriented themselves and moved toward the cathode (Movie 1 shows planarian behavior at 2 V) and spent >50% of the experimental time in the cathode-containing quadrants (Dunnett's test, *P*<0.001; Table 1). Planarians did not spend significantly more time moving toward the first cathode at 1.5 V but did so at 2 V and higher voltages (Dunnett's test, *P*=0.16 for 1.5 V, *P*=0.006 for 2 V, *P*<0.001 for 3 V and 4 V; Table 1). Once the polarity was swapped, planarians had a longer distance to travel to move toward the new cathode because they predominantly started in the most distant quadrant, at cathode 1 (Fig. 1D). We observed that following the polarity swap, the time spent moving toward cathode 2 (*f*_mov-2_) appeared to be a more consistent and sensitive measure of cathode preference for all voltages >1.5 V than the time spent at the cathode (*f­*_cat-2_) (Table 1; Fig. S2). This may be because planarians require longer to arrive at the second cathode from the most distant quadrant, causing time spent at the second cathode to be artificially low, whereas the time spent moving toward the cathode is relatively unchanged after the polarity swap.

**Table 1.**
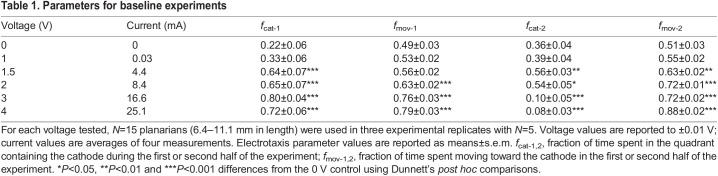
Parameters for baseline experiments

Although planarians exhibited electrotaxis at 3–4 V, they also exhibited vigorous head turning and oscillatory behavior (Movie 2 shows planarian behavior at 4 V). These behaviors caused the planarians to move more slowly toward the cathode. They still were able to reach the first cathode because they only had to traverse half of the trough, but failed to reach the cathode after the polarity switch because they needed to travel the whole distance and the adverse effects increased over time. Because of these adverse effects at higher voltages, we conducted all further experiments at 2 V.

Next, we investigated whether the planarians sensed the electric field directly or whether they reacted to secondary effects induced by the field, such as gradients in pH, temperature and convective currents, which can affect planarian behavior (Inoue et al., 2014; Ross et al., 2018; Sabry et al., 2020). We did not find significant effects of any of these factors (see Materials and Methods; Fig. S1). Thus, planarian movement toward the cathode was a direct response to the electric field.

To determine whether planarians responded to electrical current or to voltage, we tested their response to 4 V in either IO or ultrapure (MQ) water. As the *f­*_cat-2_ parameter is not indicative of electrotaxis ability at 4 V as seen in Table 1, we calculated electrotaxis parameters without an electrical polarity swap. At 4 V, the currents across a single trough of IO and ultrapure water were measured to be 25.1 mA and 3.3 µA, respectively (averaged over four measurements). In IO water at 4 V, planarians spent significantly more time in the quadrant containing the cathode (Fig. S1C) as well as spent more time moving toward the cathode compared with at 0 V (Fig. S1D). The same trend was observed when planarians were placed in MQ water (Fig. S1C,D), demonstrating that planarian movement toward the cathode is not due to the electrical current (which differed by four orders of magnitude) but due to voltage. We refer to this behavior as cathodic electrotaxis in subsequent sections.

### Planarian body length affects cathodic electrotaxis ability

**Fig. 2.**
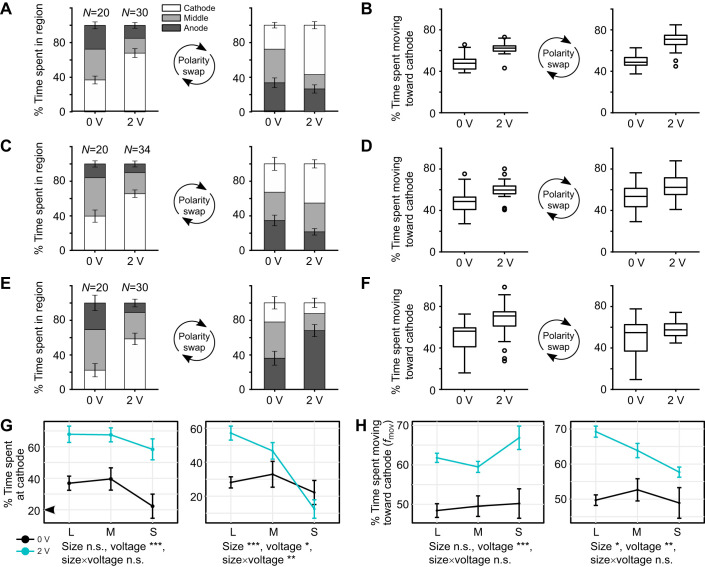
**Size and electrotaxis ability.** (A,C,E) Segmented bar plots showing the percentage of the experiment time, before and after the electrical polarity swap, spent in the cathode quadrant, anode quadrant and middle two quadrants for (A) large-, (C) medium- and (E) small-sized planarians. Error bars denote s.e.m. (B,D,F) Box-and-whisker plots showing the percentage of experiment time, before and after the electrical polarity swap, spent moving toward the cathode for (B) large-, (D) medium- and (F) small-sized planarians. Boxes indicate the 25th–75th percentiles, whiskers show the non-outlier upper and lower extremes, and the median is marked with a line. Open circles denote outliers. (G) Interaction plots showing the percentage of the time spent at the cathode for small (S), medium (M) and large (L) planarians at 0 and 2 V, before and after electrical polarity swap. (H) Interaction plots showing the percentage of the time spent moving toward the cathode for small, medium and large planarians at 0 and 2 V, before and after electrical polarity swap, and the effect of planarian size (length) and voltage. n.s., *P*>0.05; *P<0.05; **P<0.01; ***P<0.001. Data are shown as means±s.e.m.

It has been previously shown that planarian behaviors such as locomotor velocity can be size dependent (Talbot and Schötz, 2011). To determine whether size also plays a role in planarian cathodic electrotaxis, experiments were run at 2 V on *N*=94 planarians that ranged in size from 2.0 to 12.4 mm. Planarians were classified as either ‘small’, ‘medium’ or ‘large’ (Fig. S2B). Planarians in the large size class (7.6–12.4 mm) spent significantly more time at 2 V than at 0 V moving toward and staying in the quadrant containing the cathode, before and after the electrical polarity swap (Tukey’s *post hoc* test, *P*<0.001, Fig. 2A,B). Planarians in the medium size class (4.6–7.5 mm) spent significantly more time at 2 V than at 0 V moving toward and staying near the first cathode (Tukey’s post *hoc* test, *P*<0.003, Fig. 2C,D). After polarity reversal, medium-sized planarians spent significantly more time moving toward the second cathode when voltage was applied (Tukey’s *post hoc* test, *P*<0.001), although the time spent in the quadrant containing the second cathode was not significantly different between 0 and 2 V (Tukey’s *post hoc* test, *P*=0.077, Fig. 2C).

Small planarians (2.0–3.5 mm) spent significantly more time moving toward and staying near the first cathode at 2 V than at 0 V (Tukey’s *post hoc* test, *P*<0.001, Fig. 2E,F). After the electrical polarity swap, small planarians spent significantly more time moving toward the second cathode (Tukey’s *post hoc* test, *P*=0.011) but there was no difference in the time spent in the quadrant containing the second cathode (Tukey’s *post hoc* test, *P*=0.228, Fig. 2E,F). These size-dependent differences in electrotaxis behavior are summarized in Fig. 2G,H. This difference in behavior for smaller versus larger planarians was not due to differences in motility. Although smaller planarians are known to move slower than larger planarians (Hagstrom et al., 2015) and we observed differences in speed [1.70±0.05 mm s^−1^ for large planarians and 1.02±0.04 mm s^−1^ for small planarians (means±s.d.); *N*=30 and 25, respectively], there was sufficient time (90 s) for small planarians to travel the length of the trough (60 mm).

However, in contrast to large planarians, small planarians did not move toward the new cathode after the polarity swap but wandered around the anode-containing quadrant (Fig. 2E; Fig. S2C). Thus, small planarians did not exhibit the same electrotaxis behavior as large planarians. Besides differences in absolute size, we also found that differences in head size to body size existed between small and large planarians (Fig. S3).

To determine the relationship between absolute size, relative head size and the observed behavioral differences, and to characterize the role of specific anatomical structures, we took advantage of the regenerative abilities of planarians and compared electrotaxis in head, tail and trunk fragments of various sizes.

### Cathodic electrotaxis is a brain-independent behavior

**Fig. 3.**
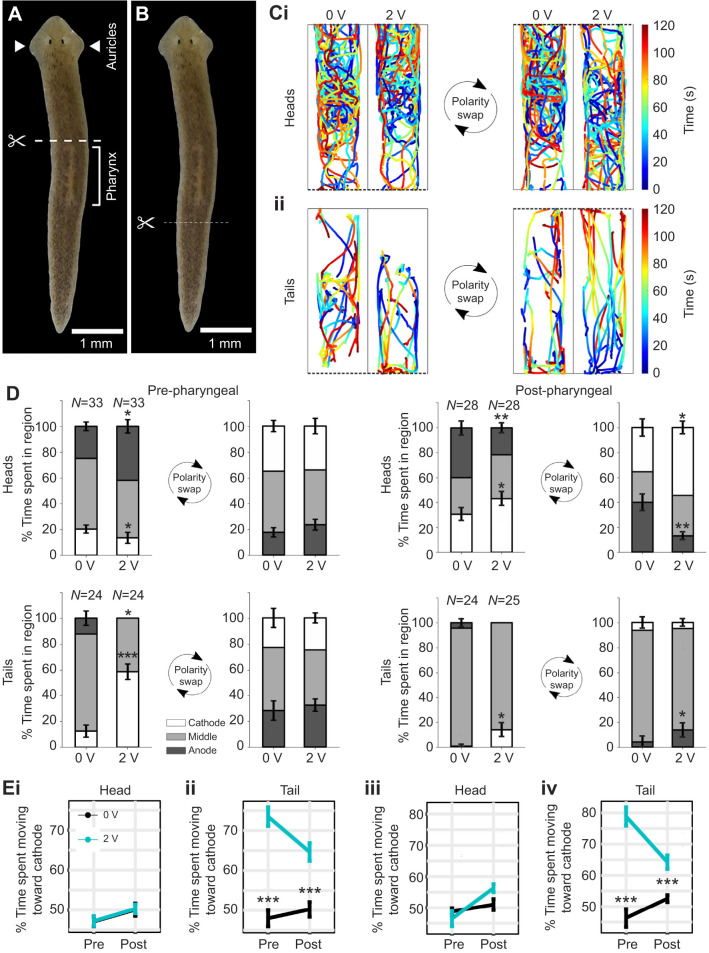
**Electrotaxis behavior of head pieces but not of tail pieces depends on cut location.** (A) Schematic showing the site of pre-pharyngeal amputations. Pharynx location indicated by bracket; auricles indicated by white arrows. (B) Schematic showing the post-pharyngeal cut location. (C) Paths traveled for a subset of *N*=15 planarians with pre-pharyngeally cut (top) heads and (bottom) tails exposed to a 0 or 2 V electric field. Dashed lines denote the location of the cathode. Heads moved randomly at both 0 and 2 V, whereas tails showed a preference for the cathode-containing quadrant. (D) Segmented bar plots showing the percentage of the experiment time, before and after the electrical polarity swap, spent in the cathode quadrant, anode quadrant and middle two quadrants. Error bars denote s.e.m. (E) Comparisons of time spent moving toward the cathode for pre- and post-pharyngeal head and tail fragments (i,ii) before polarity swap and (iii,iv) after polarity swap. *P<0.05, **P<0.01 and ***P<0.001, from the respective 0 V controls as calculated using Tukey’s *post hoc* comparisons. Tukey’s *post hoc* test, *P*=0.378 for pre-pharyngeally amputated pieces, *P*=0.063 for post-pharyngeally amputated pieces. Data are shown as means±s.e.m.

To test whether cathodic electrotaxis requires key anatomical structures, such as the head, tail, auricles and pharynx, we bisected planarians into head and tail pieces either anterior to (pre-) or posterior to (post-) the pharynx (Fig. 3A,B). Amputated planarians were allowed 1 day to heal before assaying for electrotaxis ability. If electrotaxis ability was solely size dependent and not influenced by other factors, we would expect to find that larger fragments electrotax more robustly than smaller ones, independent of their head or tail identity. However, we found that tail pieces retained the ability to electrotax independent of size (Tukey’s *post hoc* test, *P*<0.001; Fig. S3A), whereas head pieces did not exhibit cathodic electrotaxis regardless of amputation location (Fig. 3C–E; Fig. S3A). Moreover, the time-colored trajectories of head and tail pieces (Fig. 3C) show that the most striking behavioral difference between heads and tails occurs after the polarity swap, when the first cathode becomes the new anode, and the pieces need to traverse the entire trough to reach the new cathode. Although the head trajectories looked similar at 0 and 2 V, the tail trajectories were distinctively different, with straighter trajectories at 2 V that began at the new anode and ended at the new cathode, demonstrating electrotaxis.

As planarian tail pieces lack brains yet still maintain the ability to electrotax, these results demonstrated that neither the planarian brain nor the auricles were required for electrotaxis. Furthermore, because tail pieces with and without the pharynx electrotaxed (Fig. S4), these experiments showed that the pharynx is not required for cathodic electrotaxis. Moreover, small post-pharyngeal tails electrotaxed more robustly than larger pre-pharyngeal heads (Fig. 3E).

Because tail fragments, especially smaller ones, exhibited lower motility compared with head fragments, which affected the time spent at the cathodes (see Materials and Methods; Fig. 3C,D), and because the behavior at the cathodes was also influenced by other factors, such as the wall preference behavior of the planarians (Akiyama et al., 2015), the time spent at the cathode was a less suitable parameter to assay electrotaxis than the motility parameters *f*_mov-1,2_*.* Because behavioral differences were the most pronounced after the polarity swap (Fig. 3C–E), when planarians needed to traverse the entire trough to get to the cathode, we focused on *f*_mov-2_ for all further analyses.

### The relative head:body size ratio affects electrotaxis

Our experiments on cut planarians showed that tail fragments exhibited electrotaxis independently of their size, whereas size affected the electrotaxis ability in head fragments, with larger fragments (post-pharyngeally cut heads) retaining some electrotaxis ability, but smaller fragments (pre-pharyngeally cut heads) not exhibiting electrotaxis (Fig. 3). Given these data and the observed size effects in intact planarians (Fig. 2; Fig. S2), we hypothesized that the relative size of the head to the size of the body (i.e. the total size) may affect electrotaxis.

**Fig. 4.**
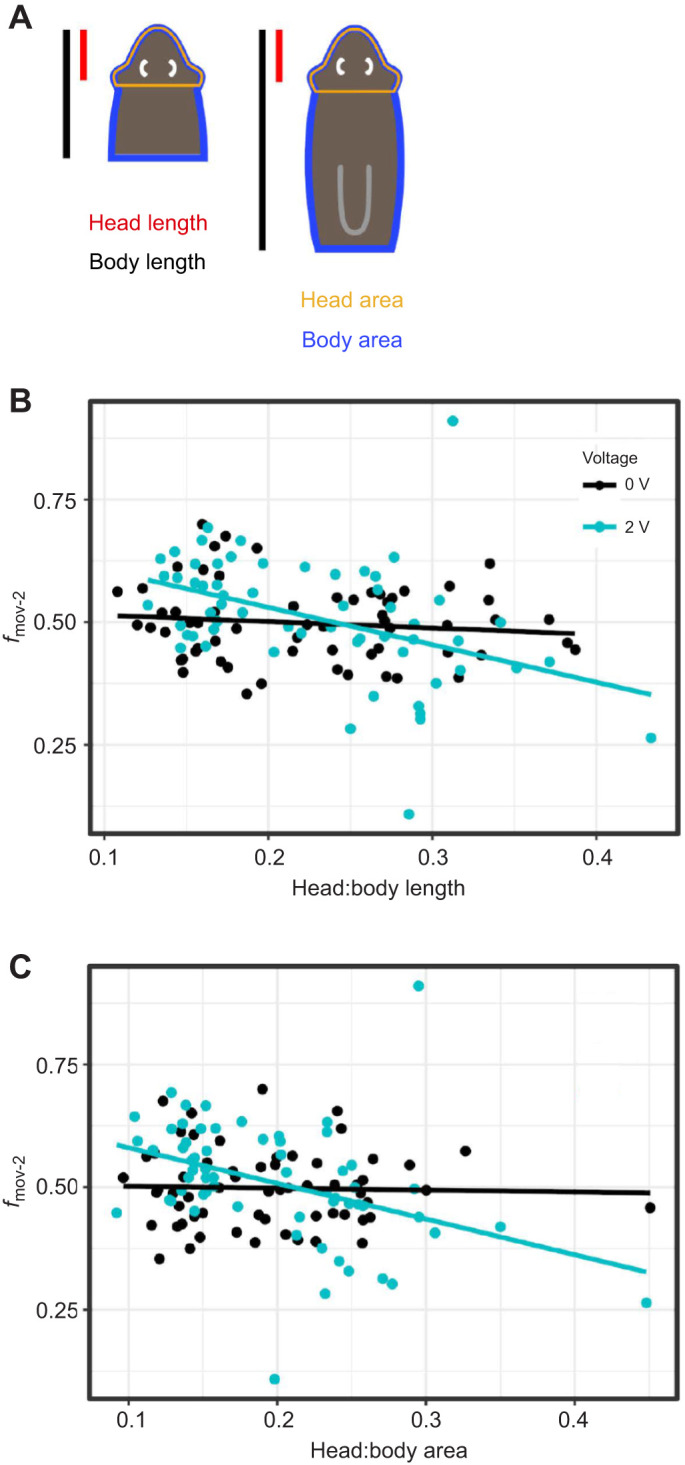
**Head size/body size proportion affects electrotaxis behavior.** (A) Schematic showing pre-pharyngeal (left) and post-pharyngeal (right) head fragments and calculation of the head size/body size proportion. For length, the red (head) and black (body) lengths were measured. For area, the outlined regions (orange, head; blue, body) were measured. (B) Interaction plots between head length to body length ratio and fraction of time spent moving toward the second cathode (*f*_mov-2_). (C) Interaction plots between head area to body area ratio and fraction of time spent moving toward the second cathode (*f*_mov-2_). Both plots show increased movement toward the second cathode with a decrease in head/body ratio at 2 V but not at 0 V, with the effect being more pronounced for area ratios.

To investigate a possible relationship between electrotaxis ability and head size/body size, we took head length, body length, head area and body area measurements and calculated head size/body size for both metrics for the pre-pharyngeally and post-pharyngeally cut heads (Materials and Methods; Fig. 4A). We found a significant interaction between the head size/body size proportion and time spent moving toward the second cathode (*f*_mov-2_) for both metrics at 2 V but not at 0 V (Fig. 4B,C). Head fragments with relatively smaller heads showed stronger electrotaxis, supporting the hypothesis that the relative size of the head affects electrotaxis ability.

Taken to the extreme, these data suggest that removal of the head should be able to restore electrotaxis in a head fragment that cannot electrotax. Thus, we dissected the role of the head for the electrotaxis ability of individual planarians.

### Head removal restores electrotaxis behavior in planarian fragments

**Fig. 5.**
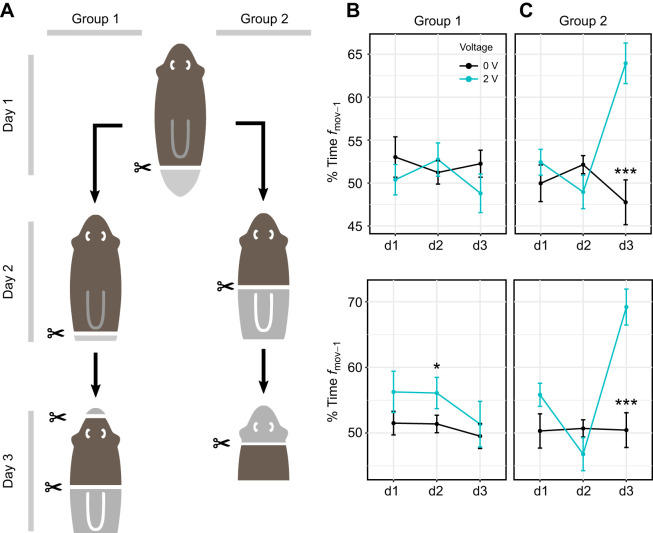
**Electrotaxis metrics are dependent on the presence or absence of a head.** (A) Schematic of experimental procedure. Notably, the indicated cuts were performed the day prior to the experimental day indicated in Materials and Methods, ‘Amputation experiments’. (B) Time spent moving toward the cathode on each day, group 1. (C) Time spent moving toward the cathode on each day, group 2. *P<0.05, **P<0.01 and ***P<0.001 from the respective 0 V controls as determined by *post hoc* comparisons. Data are shown as means±s.e.m.

First, we quantified electrotaxis in pre-pharyngeal heads and trunks (Fig. S5). These animals were exposed to a 2 V electric field for 240 s with a polarity swap at 120 s. We then decapitated the heads, removed an equivalent tissue fragment from the anterior end of the trunks, and re-evaluated electrotaxis after 24 h, to allow for healing. We found that pre-pharyngeal heads did not electrotax, but this ability was restored by decapitation (Fig. S5). Because one could argue that (1) the planarians may have differed in their ability to electrotax from the beginning and (2) any anterior cut may restore electrotaxis, we repeated this experiment using successive cuts on large planarians and tracking individual animals that we verified to have electrotaxis ability. Planarians were cut post-pharyngeally, allowed to heal for 24 h, and split into two groups (Fig. 5A). Subsequently, group 1 had a small amount of tissue removed from the posterior end and group 2 was cut pre-pharyngeally. On day 2, both groups were again assessed for electrotaxis, after which group 1 was cut pre-pharyngeally and had a small amount of tissue removed from the tip of the nose, whereas group 2 was decapitated. On day 3, both groups were assessed for a final time (Fig. 5A). Members of group 1 retained a head throughout the experiment, whereas members of group 2 lost their head in the third amputation. Each group received the same number of posterior and anterior cuts, to account for any changes that may be introduced by amputation.

We found that post-pharyngeally cut heads (day 1) showed no electrotaxis, consistent with earlier experiments. Within group 1, post-pharyngeal heads with an additional posterior wound (day 2) showed weak electrotaxis. Pre-pharyngeal amputation coupled with an anterior wound (day 3) caused loss of electrotaxis (Fig. 5B). Similarly, within group 2, pre-pharyngeal heads (day 2) failed to show electrotaxis. However, trunk pieces formed by subsequent decapitation (day 3) showed clear recovery of electrotaxis (Movie 3) with statistically significant changes in both *f*_mov-1_ and *f*_mov-2_ (Fig. 5C). A non-decapitating anterior wound (group 1) did not restore electrotaxis. Based on these results, we conclude that it is the presence or absence of a head that is the strongest factor determining planarian cathodic electrotaxis.

## DISCUSSION

Using an automated experimental setup that minimizes experimenter bias and other external influences, our data show that the apparent electrotactic response by planarians is in fact due to the electric field rather than to other environmental cues. This is an important distinction to make as planarians are known to sense temperature and chemical gradients (Inoue et al., 2014, 2015), and electric fields in water can generate thermal, pH and convective effects (Gunji and Washizu, 2005; May and Hillier, 2005). Although these ancillary effects of the method used to generate static electrical fields should be minimal given the small voltage applied, it was important to test for them as it was unclear how sensitive planarians are. The voltages used in previous studies (Pearl, 1903; Hyman and Bellamy, 1922; Robertson, 1927; Fries, 1928; Hyman, 1932), whether directly reported or estimated based on current and arena dimensions (Table S1), were much larger than the 2 V we used in our experiments, and were less likely to have isolated the effects of the electric field from other associated environmental changes.

The high and variable voltages involved in previous studies likely account for some of the variability in reported results, as well as observed dramatic behaviors such as planarians curling up on their sides (Hyman and Bellamy, 1922; Hyman, 1932), scrunching (Robertson, 1927), paralysis and death (Pearl, 1903). These prior studies largely assumed that the response was elicited by the current (Pearl, 1903; Hyman and Bellamy, 1922; Robertson, 1927; Fries, 1928; Hyman, 1932) (Table S1). The finding that planarians electrotax similarly in both ionized and deionized water despite a current difference of four orders of magnitude demonstrates that *D. japonica* planarians sense and respond to voltage and not to electrical current. The distinction between voltage and current informed our subsequent experimental design and is key to future efforts to determine the mechanism underlying planarian electrotaxis.

We showed that electrotaxis is not tied to a specific anatomical structure via amputation experiments. We assayed the role of the brain and sensory structures such as the auricles and pharynx, which are used in chemotaxis (Asano et al., 1998; Miyamoto et al., 2020). Pre-pharyngeally cut tail fragments lack the brain and auricles, whereas post-pharyngeally cut tail fragments lack the brain, auricles and pharynx. The presence of electrotactic behavior in both types of tail fragments shows that the brain, pharynx and auricles are not required. Thus, voltage sensing, and subsequent directed motion cannot be attributed to specific anatomical structures but rather depends on a broadly distributed or graded property throughout the body. In addition, our voltage sweep showed that cathodic electrotaxis does not result from direct electrical action on either the cilia or muscles, because we were able to elicit the behavior in planarians gliding (1.5–2 V) and using musculature-driven locomotion (scrunching) (3–4 V).

Our observation that electrotaxis is weaker in smaller worms is interesting, as smaller planarians do not represent a different life stage during which certain structures or tissues might be absent or immature. However, our results from varying sizes of both intact planarians and fragments show that electrotaxis ability is not a direct consequence of size (Fig. S3A). Instead, we found that differences in head size to body size existed between small and large planarians (Fig. S3B,C) and that the relative size of head to body correlated with electrotaxis ability (Fig. 4). Strikingly, a fragment containing a head lacked the ability to electrotax, whereas a similarly sized fragment without a head retained this ability. This finding is the opposite of what was reported in the literature for other planarian species (Table S1), wherein it was found that head pieces in an electric field behaved more like intact planarians than other fragments (Pearl, 1903; Fries, 1928).

Behavioral differences among species are known to exist for other stimulated behaviors (Ireland et al., 2020) and it is possible that the electrotaxis response of *D. japonica* differs from the other planarian species previously studied. An alternative explanation is that our use of lower field strength to avoid the adverse effects of electric field exposure described in the literature (scrunching, curling, paralysis and death; Pearl, 1903; Hyman and Bellamy, 1922; Robertson, 1927; Hyman, 1932) elicited more differentiated behaviors.

**Fig. 6.**
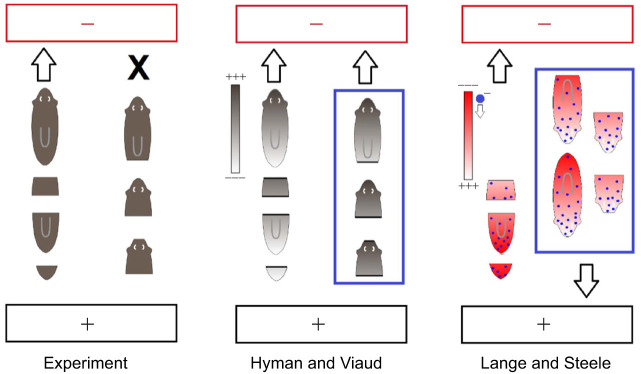
**Testing proposed models against experimental data from the present study.** The cathode is indicated in red and the anode in black. Left: results obtained in our experiments. Cathodic electrotaxis is indicated by an arrow, lack of electrotaxis is indicated by X. Middle: the Hyman and Viaud models (Hyman, 1932; Viaud and Medioni, 1951; Viaud, 1952a); partially explain the data but predict cathodic electrotaxis of head fragments, which was not observed in our experiments. Right: the Lange and Steele models (Lange and Steele, 1978) partially explain the data but predict anodic electrotaxis of intact planarians and head fragments, which was not observed in our experiments. The blue boxed cases highlight which experimental data are not explained by each model.

Previous work on planarian electrotaxis has attributed the reaction of the worms to electric fields to direct action of the electric current on the muscles (Pearl, 1903; Fries, 1928), intrinsic bioelectric gradients of the animal (Robertson, 1927; Hyman, 1932; Lange and Steele, 1978) or head-to-tail differences in electrical conductance (Viaud and Medioni, 1951; Viaud, 1952a). Lange and Steele (1978) proposed an electrochemical model for axial patterning and measured the intrinsic bioelectric gradient. They reported that the head was negatively charged relative to the body, with a posteriorly increasing positive charge toward the tail. Based on these data, they proposed that the head-to-tail bioelectric gradient caused electrophoresis of a negatively charged head inhibitor molecule that is produced in the brain. Thus, according to their model, there exists a static bioelectric gradient superimposed by a dynamic concentration gradient of a negatively charged morphogen that travels head to tail. Upon decapitation, a piece with its bioelectric gradient aligned with an external electric field would thus experience a positively charged anterior relative to its posterior and migrate toward the cathode, as observed in the classical patterning experiments by Marsh and Beams (1952) (Fig. 6).

Conversely, Hyman (1932) proposed that the bioelectric gradient correlates with a metabolic gradient and, because the head was more metabolically active, the head region was positively charged compared with the body (Fig. 6). Recent work (Durant et al., 2017) using the DiBAC4(3) voltage reporter (Oviedo et al., 2008) showed that the very tip of the head region is relatively depolarized (positively charged). Although this result seems to support Hyman's model, it does not directly contradict the measurements of Lange and Steele (1978), given the coarse nature of their measurements and the observation that most of the head does not appear depolarized in the DiBAC experiments. DiBAC experiments also showed that trunk pieces have polarity, with anterior wounds being more positively charged than posterior wounds (Durant et al., 2019), in agreement with both model predictions and the observed cathodic electrotaxis (Marsh and Beams, 1952).

A fourth explanation for electrotaxis was provided by Viaud and Medioni (1951), who reported that electrical conductance and excitation was consistently greater and the threshold for a response to current was lower when the head of the planarian was facing the cathode than when it faced the anode. This observation was reproduced in head and tail fragments (Viaud, 1952a). Thus, this model makes similar behavioral predictions as the Hyman model.

How do these different explanations perform in the light of our experimental data? We can rule out the direct action of current on the musculature as the driving force for electrotaxis because we were able to elicit electrotaxis at 2 V without musculature-driven locomotion. The models that propose anterior–posterior bioelectric or conductance gradients similarly cannot explain all the data (Fig. 6).

Although all models can explain the observed cathodic electrotaxis of trunk and tail fragments, the Hyman and Viaud models would predict head fragments to move equally toward the cathode, which was not observed in our experiments. The Lange and Steele model would predict intact planarians and head fragments to move toward the anode, given the presumed negative charge of the planarian head and constant production of a negatively charged morphogen in the head; however, this was also not observed in our experiments. Thus, none of the current models can explain all our data.

One may question why we see electrotaxis in intact planarians but not in post-pharyngeally cut head fragments. This can be explained by the difference in head to body ratio, which we have shown affects electrotaxis ability (Fig. 4). What distinguishes the head from the rest of the planarian body is the presence of a brain consisting of many different types of neurons organized in a bilobed neuronal network (Cebrià et al., 2002; One Pagan, 2014). Viaud (1952a) already proposed that differences in head and tail current sensitivity and excitation anisotropy may result from the quantity and type of neurons in each fragment, and suggested that the animal orients itself in the electrical field to maximize neuronal excitation. In trunk and tail fragments, the ventral nerve cords run parallel to the anterior–posterior axis, and thus could promote alignment with the external field. In contrast, neuronal connections in the head extend in all directions (as seen from the center of the head); thus, there may not be a preferred direction of orientation and no electrotaxis is observed. Although it is possible that the commissures (smaller bundles of nerves that branch off perpendicular to the ventral nerve cords) also play a role in electrotaxis, our data show that if commissures play a role, their effect does not seem to dominate the response; else we would expect head fragments to also electrotax, as they contain many commissures.

The finding that planarian electrotaxis is a brain-independent behavior differs from electrotaxis in other invertebrates, in which it is mediated by specific neurons in the head. The nematode *Caenorhabditis elegans* moves toward the cathode in response to electric fields (Shanmugam, 2017), and this behavior is disrupted when amphid sensory neurons in the head ganglia are surgically severed (Gabel et al., 2007; Salam et al., 2013; Chrisman et al., 2016). In *Drosophila* larvae, a subset of peripheral neurons in the terminal organ at the anterior tip of the head becomes strongly activated when the neuronal axis becomes aligned with the direction of electric field (Riedl, 2013). In contrast, our results show that neurons in the head are not required for planarian electrotaxis; instead, their presence seems to impair the behavior. The quantitative data and methods presented here lay the foundation for future studies to dissect how headless planarian fragments sense electric fields, and to determine how inhibitory signals from the head impair cathodic electrotaxis.

## Supplementary Material

Supplementary information
