# Supplementary material for: Head removal enhances planarian electrotaxis
Source: J Exp Biol. 2022 Sep 7;225(17):jeb243972. doi: 10.1242/jeb.243972 (PMC9482365; doi:10.1242/jeb.243972)
Supplement: Supplementary information [file jexbio-225-243972-s1.pdf]

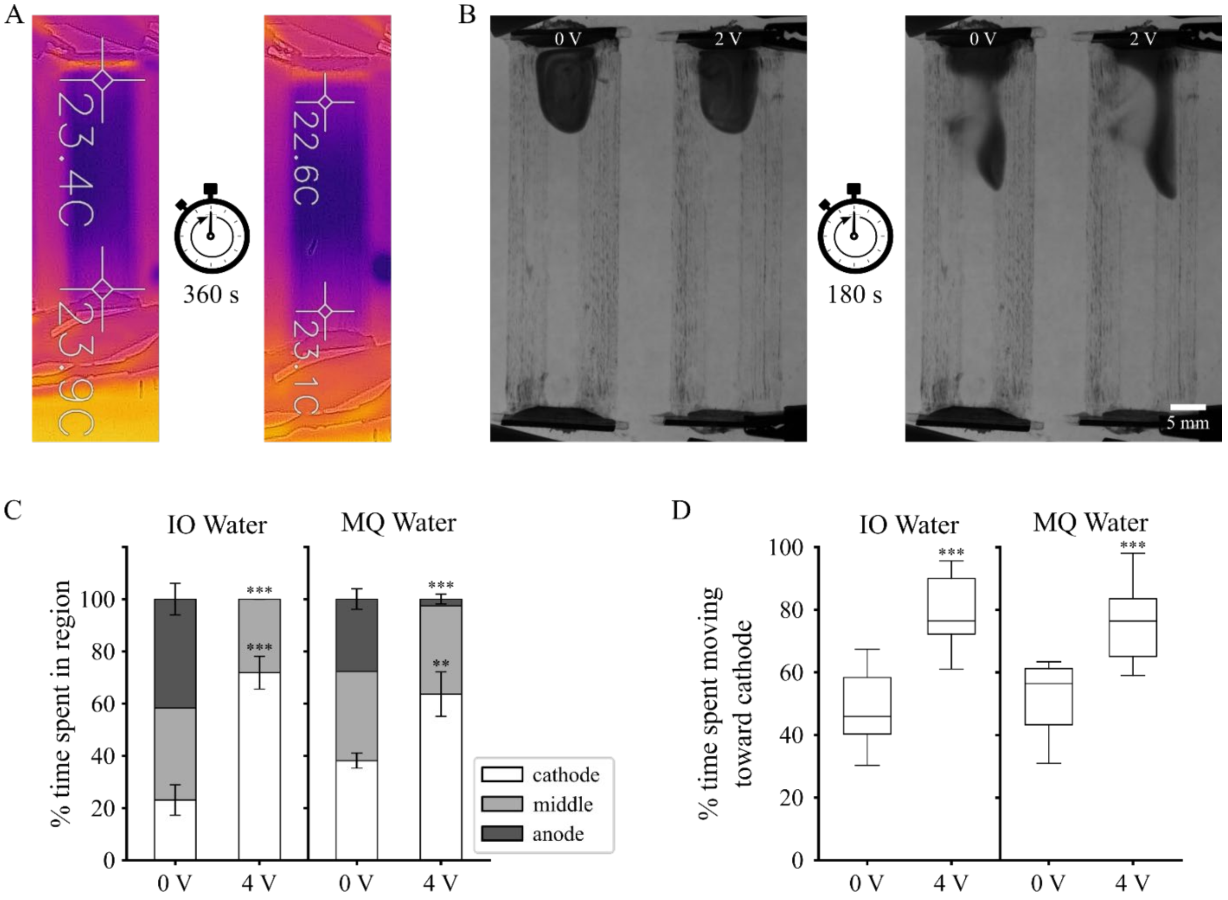

Fig. S1. Electrotaxis is not a consequence of temperature gradients, convection, or current. (A) Infrared images of a trough arena filled with 4 mL of IO water taken before (left) and after (right) a 2 V electric field was applied for 360 s. The measured temperature difference between the electrodes is 0.5C before and after, which is within the noise of the measurement. (B) Images taken of dye in trough arenas before (left) and after (right) 180 s of either 0 V or 2 V. (C) Bar graphs showing the average percent experiment time planarians spent in the cathode or anode quadrants or the middle two quadrants at 0 V and 4 V in IO or ultrapure (MilliQ; MQ) water. Error bars denote standard error. (D) Box-and-whisker plots showing the percent experiment time planarians spent moving toward the cathode. (C-D) N = 15 planarians were placed in IO or MQ water at 0 V or 4 V. ** denotes $p < 0.01$ and *** denotes $p < 0.001$ from factorial ANOVA. The interaction between water type and voltage was not statistically significant for either C or D.

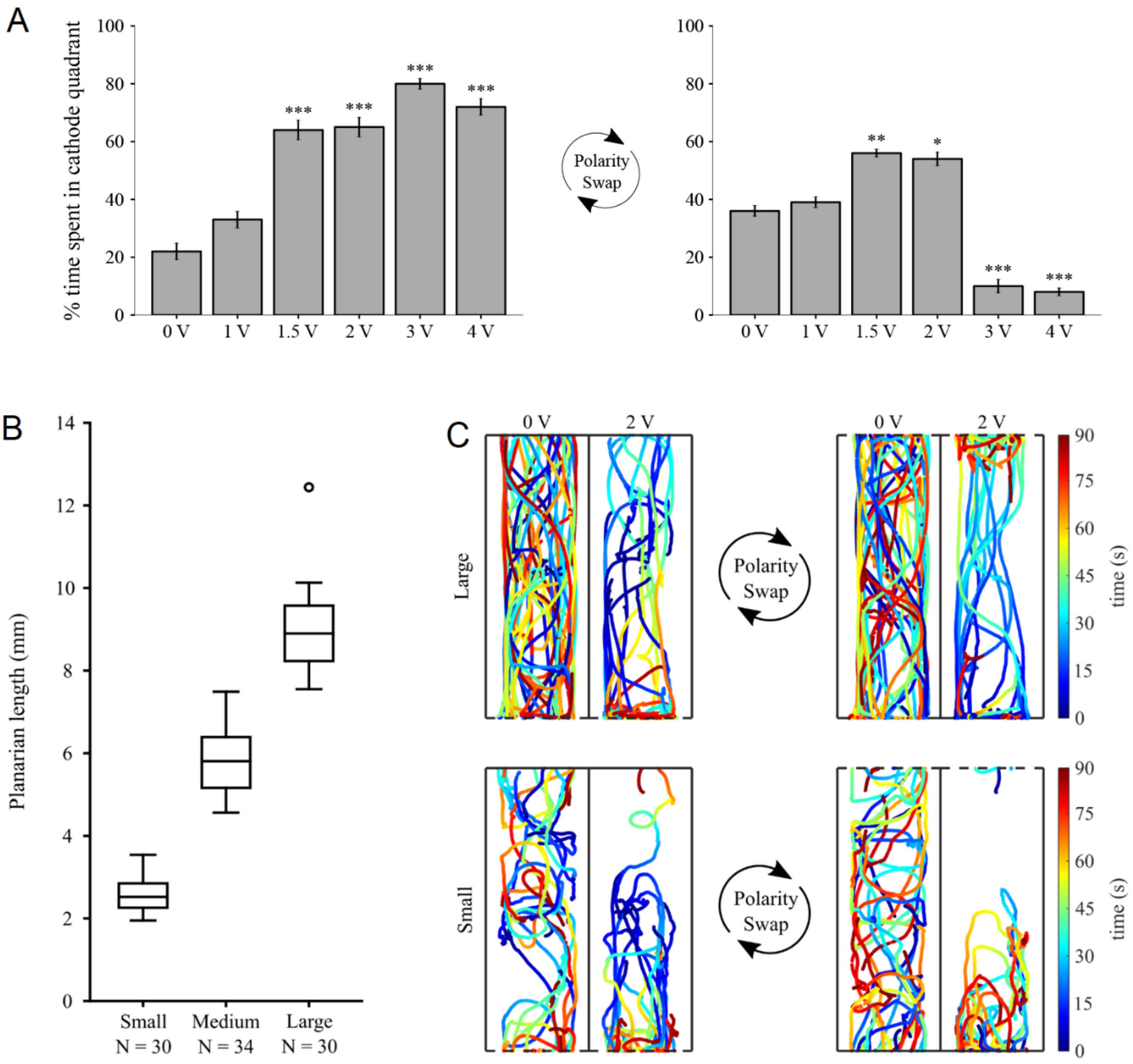

Fig. S2. Voltage and size dependence of planarian movement in the electric field. (A) Bar plots showing the percentage of experimental time spent in the quadrant containing the cathode before (left) and after (right) the electrical polarity swap. Bar height denotes mean percent experimental time and error bars denote standard error. * denotes $p < 0.05$, ** denotes $p < 0.01$ and *** denotes $p < 0.001$ differences from the 0 V control using Dunnett's post-hoc comparisons. N=15 planarians (6.4 mm – 11.1 mm in length) were used in 3 replicates with N=5 per experiment. (B) Box-and-whisker plots showing the size distributions of planarians in the small, medium, and large size classes. (C) Paths traveled for a subset of N = 15 (top) large and (bottom) small planarians exposed to a 2 V electric field for 180 s with a polarity swap occurring at 90 s. Dashed lines denote the location of the cathode electrode.

Journal of Experimental Biology • Supplementary information

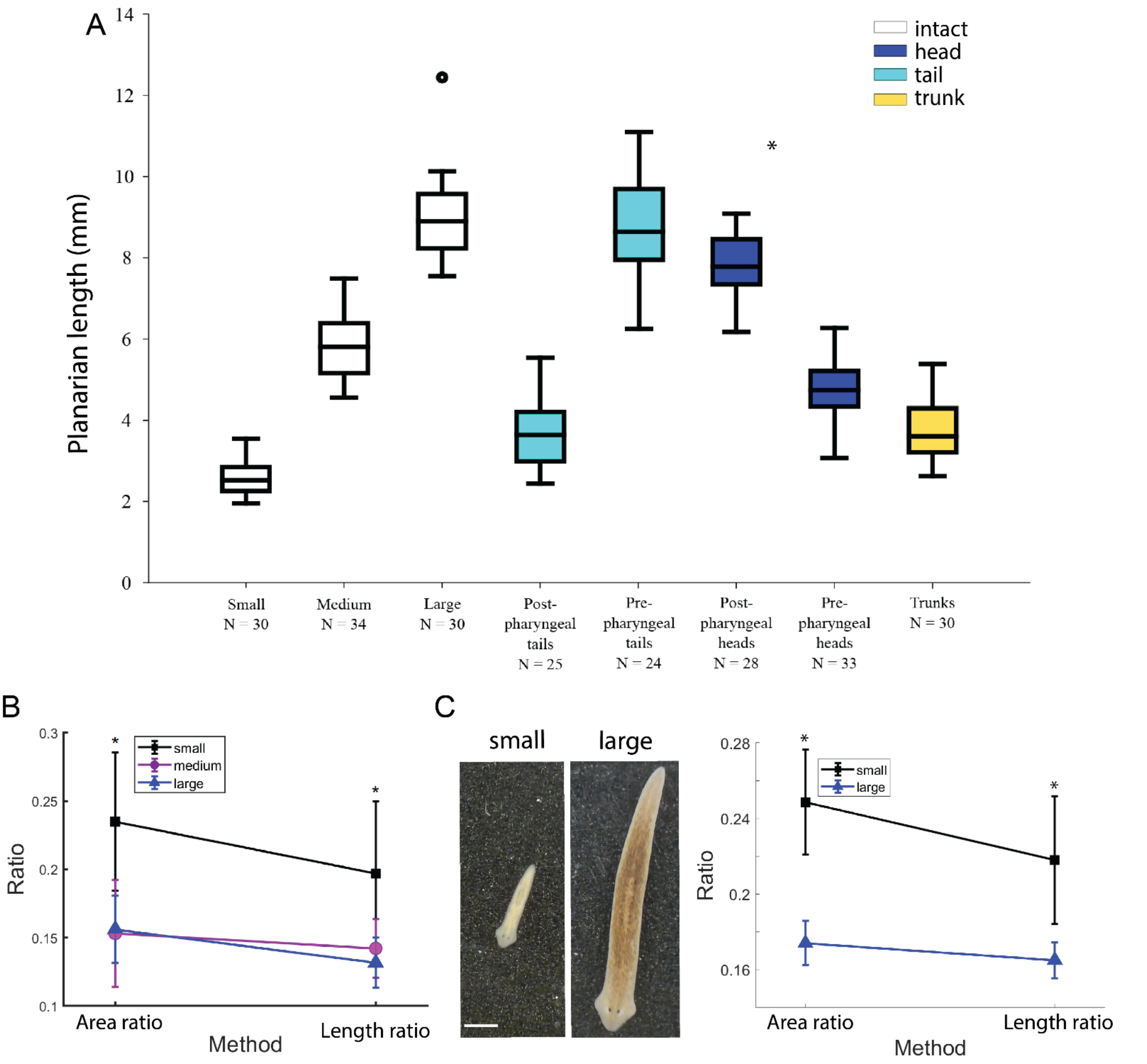

Fig. S3. Lengths and head-to-body ratios of planarians used in electrotaxis experiments. (A) Box-and-whisker plot showing the lengths of representative subsets of planarians used in the different experiments. Fragments are color coded by type. Open circles denote outliers. (B) For planarians used in electrotaxis experiments, there was a significant difference ($p < 0.05$) in head-to-body-ratio between small and medium, and small and large planarians, as measured by length and by area. There was no difference between medium and large planarians. (C) Representative images of small and large planarians from high magnification imaging, which was used to confirm the observed differences in head-to-body-ratio from the lower resolution data from the electrotaxis setup. High magnification data also shows that there is a difference between length ratios and area ratios for small but not for large planarians, implying that any comparisons using length ratios is an underestimation of the differences between the two groups. Scale bar: 1mm.

Journal of Experimental Biology • Supplementary information

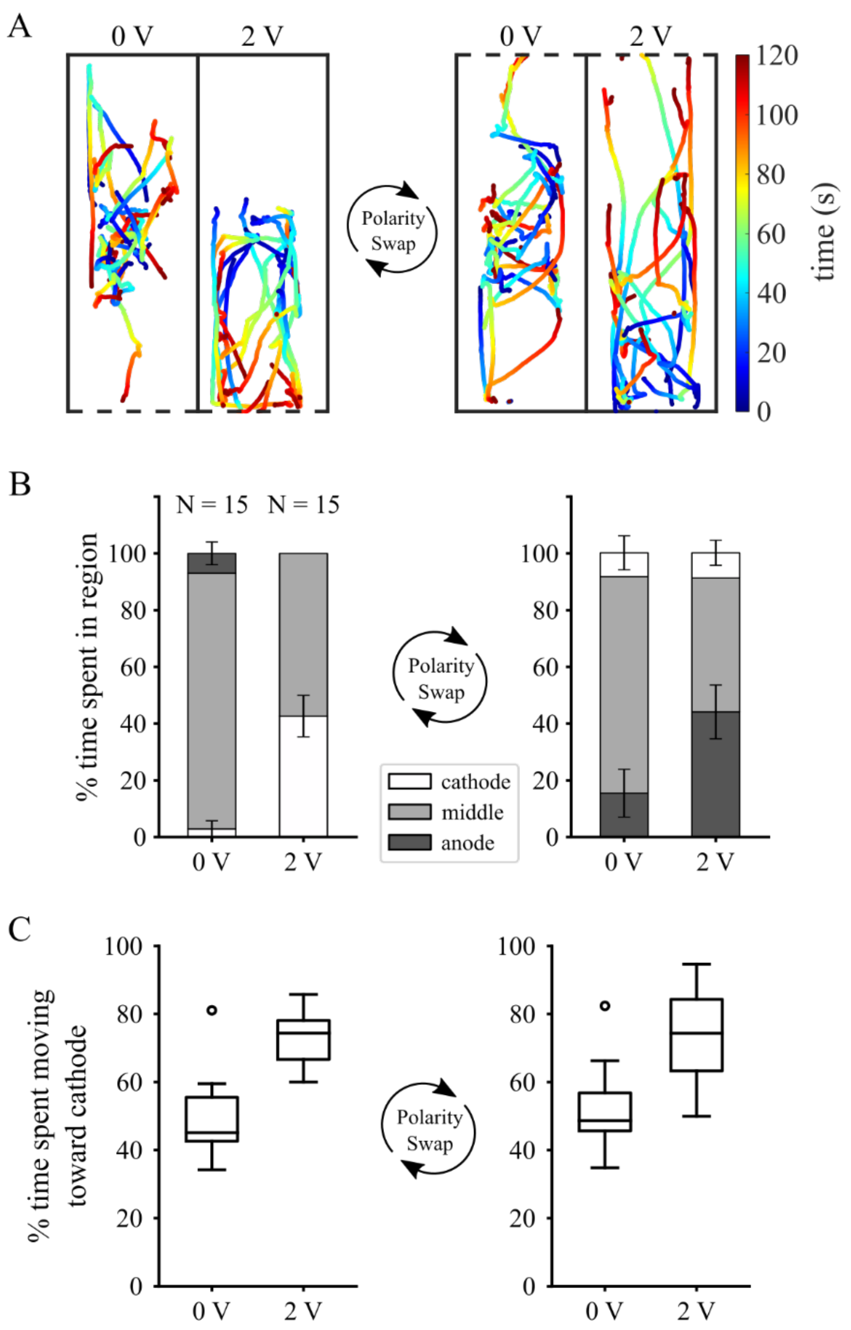

Fig. S4. Removal of the pharynx does not disrupt electrotaxis ability in pre-pharyngeally cut tail pieces. (A) Paths traveled for N = 15 pre-pharyngeally cut planarian tail pieces with pharynges removed. Dashed lines represent the location of the cathode. (B) Segmented bar plots showing the percent experiment time, before and after the electrical polarity swap, spent in the cathode quadrant, anode quadrant, and middle two quadrants. Error bars denote standard error. (C) Box-and-whisker plots showing the percentage of experiment time, before and after the electrical polarity swap, spent moving toward the cathode. Open circles denote outliers. Although 0V and 2V groups differ for all comparisons, the interaction between voltage and pharynx treatment was not significant for any (factorial ANOVA).

Journal of Experimental Biology • Supplementary information

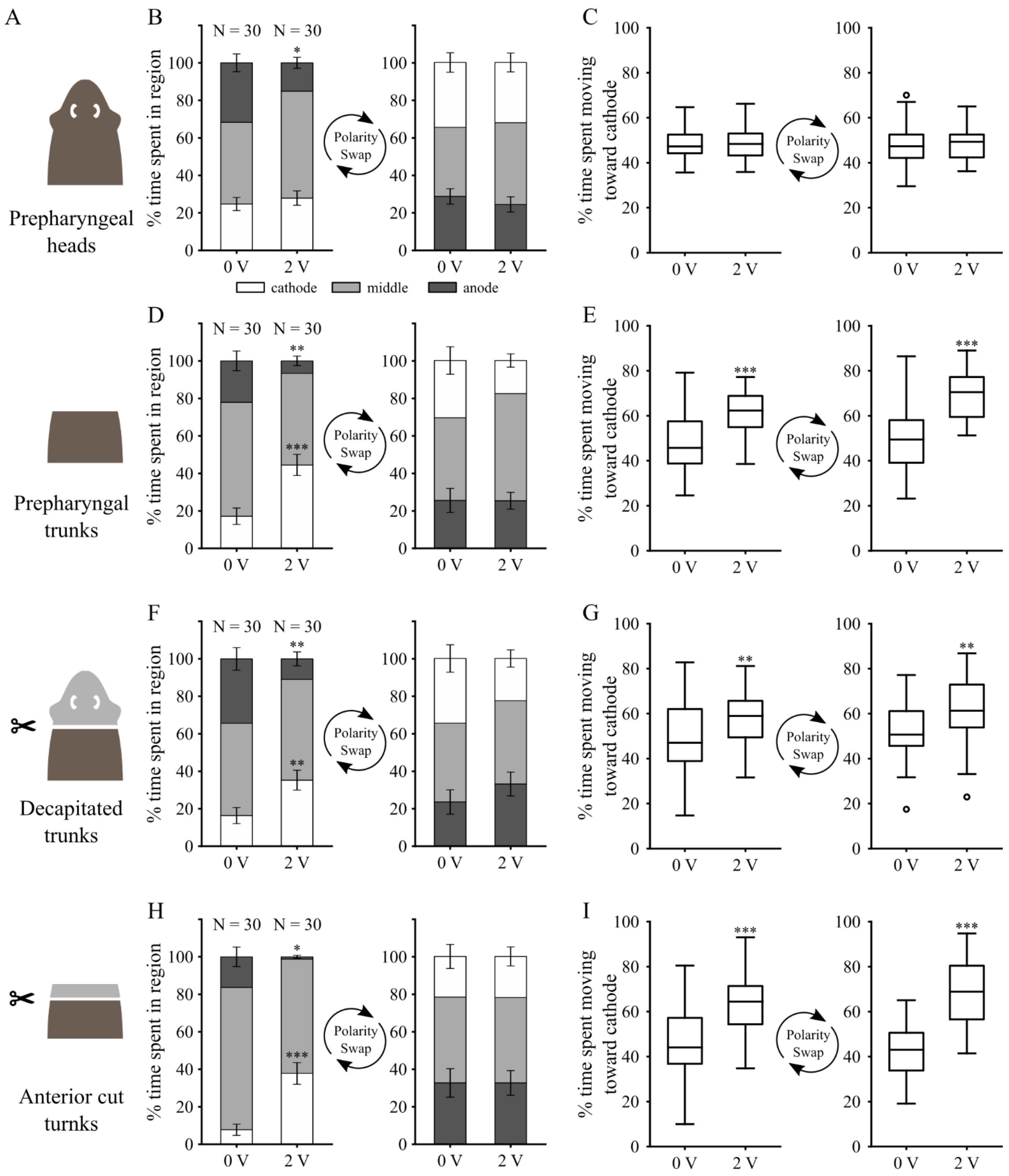

Journal of Experimental Biology • Supplementary information

Fig. S5. Decapitation of pre-pharyngeally cut head pieces restores cathodic electrotaxis ability. Animals were exposed to a 2 V electric field for 240 s with a polarity swap at 120 s. (A) Schematics illustrating planarian fragments and cutting process. The grey colored region was first removed, then the brown colored piece was tested for electrotaxis ability 24 hours later. (B, D, F, H) Segmented bar plots showing the percent experiment time, before and after the electrical polarity swap, spent in the cathode quadrant, anode quadrant, and middle two quadrants for (B) pre-pharyngeally cut head pieces (D) pre-pharyngeally cut trunk pieces, (F) pre-pharyngeally cut head pieces that have been decapitated and, (H) pre-pharyngeally cut trunk pieces that have had an anterior cut. Error bars denote standard error. (C, E, G, I) Box-and-whisker plots showing the percentage of experiment time, before and after the electrical polarity swap, spent moving toward the cathode for (C) pre-pharyngeally cut head pieces (E) pre-pharyngeally cut trunk pieces, (G) pre-pharyngeally cut head pieces that have been decapitated and, (I) pre-pharyngeally cut trunk pieces that have had an anterior cut. Open circles denote outliers. * denotes $p < 0.05$, ** denotes $p < 0.01$ and *** denotes $p < 0.001$ from the respective 0 V controls using the statistical analyses described in Methods.

Journal of Experimental Biology • Supplementary information

Table S1. Summary of existing planarian electrotaxis literature. Jenkins (Jenkins 1967) reviews several of these older studies in more detail.

Reference	Species	Electrical parameters	Amputated worms	Cathodic electrotaxis	Behavior	Proposed mechanism
(Pearl 1903)	*Planaria maculata (=Dugesia tigrina), dorotocephala, gonocephala*	n/a	Y	Observed in intact worms and anterior pieces but not tails	Head turning towards cathode, crawling (=scrunching), curling at high current	Stimulation of muscle fibers oriented parallel to the current
(Hyman & Bellamy 1922)	*Planaria maculata (=Dugesia tigrina),* Unidentified triclad	110V DC generator w/variable resistor	N	Y	Curling with ventral side, head and tail towards cathode	Bioelectric gradient resulting from metabolic gradient; with head more positive than tail
(Robertson 1927)	*Polycelis nigra*	1.5-10mA DC	Y	Y in all conditions; longitudinally cut worms and head/trunk/tail fragments	Gliding, crawling (=scrunching)	Electric gradient; same orientation as proposed by Hyman
(Fries 1928)	*Planaria maculata (=Dugesia tigrina), planaria agilis (=Dugesia dorotocephala)*	0.3-0.5mA, 5V or less	Y	Y for untreated worms and head and tail fragments, reversed with strychnine	Turning, some movement, contraction of end near electrode	Direct stimulation of nerves or muscles by current
(Hyman 1932)	*Planaria dorotocephala, Curtisia foremanii* (= *Planaria simplissima*), *Procotyla fluviatilis* (= *Dendrocoelum lacteum*)	~1mA, 5-20V	N	Y	Crawling (=scrunching), turning, U- and W-shaped curling on side	Bioelectric gradient, with head more positive than tail
(Marsh & Beams, 1952)	*Dugesia tigrina*	Current densities 1.6-24.4 $\mu A/mm^2$	Y	Y	Movement of fragments toward cathode; no intact planarians tested	Inherent polarity of cut pieces
(Viaud & Medioni 1951)	*Dugesia lugubris*	variable	Y	Y	Brief stop and head-to-tail contraction, followed by pivoting and moving to cathode	Anisotropy of electrical conductance; alignment with head toward cathode has least resistance

Journal of Experimental Biology • Supplementary information

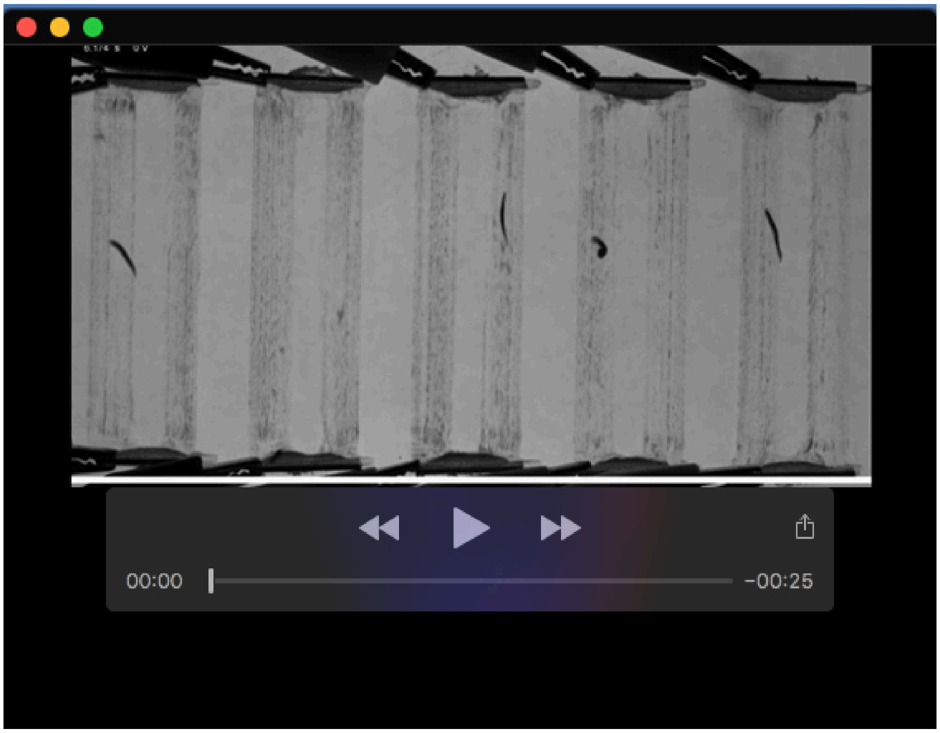

Movie 1. Planarian movement with and without electric field. Planarians move randomly in troughs when no voltage is applied but show directed movement towards the cathode when 2V is applied. Timestamp is in seconds and the white bar represents the location of the cathode. Movie is sped up 4x.

Journal of Experimental Biology • Supplementary information

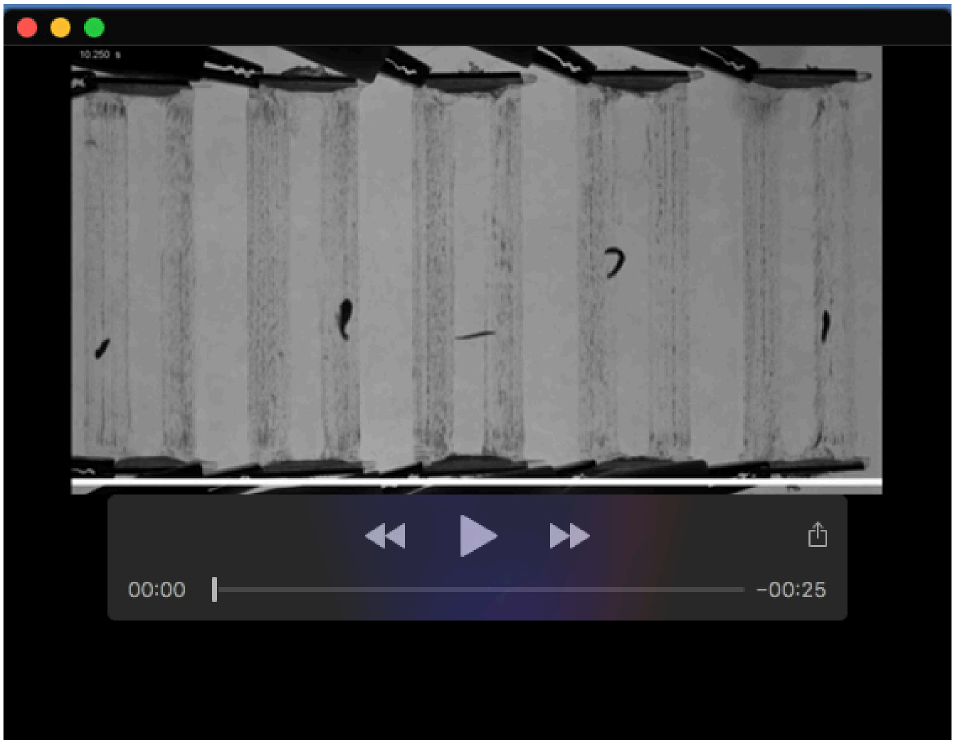

Movie 2. Planarians display muscle-driven locomotion at 4V. Planarians move randomly in troughs when no voltage is applied but show directed movement using muscle-driven locomotion towards the cathode when 2V is applied. Timestamp is in seconds and the white bar represents the location of the cathode. Movie is sped up 4x.

Journal of Experimental Biology • Supplementary information

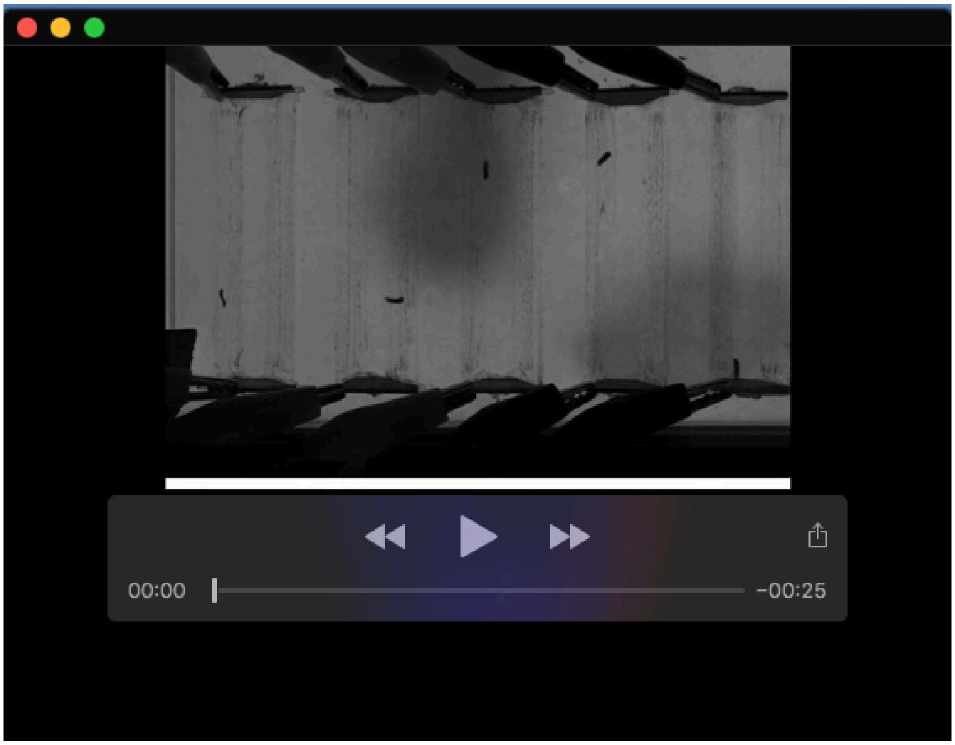

Movie 3. Head removal rescues loss of cathodic electrotaxis in head fragments. The response of head fragments to 2V is shown first, before and after polarity swap, followed by the response to 2V of the same individuals after decapitation and 1 day of healing, before and after polarity swap. The white bar represents the location of the cathode. Movie is sped up 8x.

References

Fries, E.F.B., 1928. Drug action in galvanotropic responses. *Journal of General Physiology*, 11(5), pp.507–513.

Hyman, L.H., 1932. Studies on the Correlation between Metabolic Gradients, Electrical Gradients, and Galvanotaxis. II. Galvanotaxis of the Brown Hydra and Some Non-Fissioning Planarians. *Physiological Zoology*, 5(2), pp.185–190.

Hyman, L.H. & Bellamy, A.W., 1922. Studies on the correlation between metabolic gradients, electrical gradients, and galvanotaxis. *Biological Bulletin*, 43(5), pp.313–347.

Jenkins, M.M., 1967. Aspects of Planarian Biology and Behavior. *Chemistry of Learning*, pp.117–143.

Pearl, R., 1903. The Movements and Reactions of Fresh-water Planarians: A Study in Animal Behaviour; The Quarterly Journal of Microscopical Science. *The Quarterly Journal of Microscopical Science*, 46, pp.509–714.

Robertson, J.A., 1927. Galvanotropic Reactions of Polycelis nigra in Relation to Inherent Electrical Polarity. *Journal of Experimental Biology*, 5(1), pp.66–88.

Viaud, G. & Medioni, J., 1951. Anisotropie d'excitation et variations des seuils de réaction au courant galvanique en fonction de la taille chez Planaria lugubris, O. Sch. *Comptes rendus des seances de la Societe de biologie et de ses filiales*, 145(15–16), pp.1228–31.
